# Dietary Nanoparticles Interact with Gluten Peptides and Alter the Intestinal Homeostasis Increasing the Risk of Celiac Disease

**DOI:** 10.3390/ijms22116102

**Published:** 2021-06-05

**Authors:** Clara Mancuso, Francesca Re, Ilaria Rivolta, Luca Elli, Elisa Gnodi, Jean-François Beaulieu, Donatella Barisani

**Affiliations:** 1 School of Medicine and Surgery, University of Milano-Bicocca, 20900 Monza, Italy; clara.mancuso@unimib.it (C.M.); francesca.re1@unimib.it (F.R.); ilaria.rivolta@unimib.it (I.R.); e.gnodi@campus.unimib.it (E.G.); 2 Laboratory of Intestinal Physiopathology, Faculty of Medicine and Health Sciences, Université de Sherbrooke, Sherbrooke, QC J1H 5N4, Canada; Jean-Francois.Beaulieu@usherbrooke.ca; 3 Centre for the Prevention and Diagnosis of Celiac Disease, Gastroenterology and Endoscopy Unit, Fondazione IRCCS Ca’ Granda Ospedale Maggiore Policlinico, 20122 Milan, Italy; luca.elli@policlinico.mi.it

**Keywords:** celiac disease, food additives, metallic nanoparticles, nanotoxicity, risk factors

## Abstract

The introduction of metallic nanoparticles (mNPs) into the diet is a matter of concern for human health. In particular, their effect on the gastrointestinal tract may potentially lead to the increased passage of gluten peptides and the activation of the immune response. In consequence, dietary mNPs could play a role in the increasing worldwide celiac disease (CeD) incidence. We evaluated the potential synergistic effects that peptic-tryptic-digested gliadin (PT) and the most-used food mNPs may induce on the intestinal mucosa. PT interaction with mNPs and their consequent aggregation was detected by transmission electron microscopy (TEM) analyses and UV–Vis spectra. In vitro experiments on Caco-2 cells proved the synergistic cytotoxic effect of PT and mNPs, as well as alterations in the monolayer integrity and tight junction proteins. Exposure of duodenal biopsies to gliadin plus mNPs triggered cytokine production, but only in CeD biopsies. These results suggest that mNPs used in the food sector may alter intestinal homeostasis, thus representing an additional environmental risk factor for the development of CeD.

## 1. Introduction

Nanotechnologies and different types of nanomaterials are materials studied in depth due to their unique features and diverse potential applications, from electronics to medicine. Together with the investigation of their tailored beneficial properties, the need to consider and evaluate their influence on the environment and on living organisms is growing. Currently, a wide range of diverse materials are prepared in the form of nanostructures, and the majority of studied nanomaterials are carbon-, metal-oxide-, or polymer-based particles in nano dimensions (nanoparticles) [1]. By definition, nanoparticles (NPs) include materials with dimensions of 1–100 nm, which can be in an unbound or an aggregate/agglomerated state. In the latter case, 1–50% of the particles must be in this size range with at least one external dimension or, if larger, must maintain NP properties [2].

NPs and their aggregates present different chemo-physical properties, compared to their macro-counterparts, that are useful in a wide range of applications including nanomedicine and the cosmetic and food sectors [3,4]. Therefore, consciously or not, a large amount of nanoparticles comes into contact with our bodies.

Although the literature regarding the toxicity and safety of NPs has increased exponentially in recent years, results are often controversial, and an extensive understanding of the NPs effects on human health is still lacking. In fact, interactions between NPs and biological systems are quite complex, involving several factors such as size, shape, and surface properties of NPs, cell type [5], and also the physicochemical properties of the surrounding environment [6]. Thus, NPs that are theoretically safe could become dangerous in a complex environment, such as the gastrointestinal tract, leading to unwanted pathological consequences [7,8].

Metallic nanoparticles (mNPs) were enlisted in the Nanotechnology Consumer Product Inventory as the most abundant category of nanomaterials entering through the ingestion route [9]. Different types are used in the food sector, mostly the food-whitening agent TiO2NPs, ZnO-, and Ag-NPs for their antimicrobial effects in food-contact materials, and AuNPs are used to improve plant yield and growth. Studies on the gastrointestinal tract have shown that mNPs can alter intestinal homeostasis and permeability, potentially increasing the passage of immunogenic molecules in the lamina propria and, in turn, triggering the immune system [10]. This could be relevant in patients with autoimmune disorders [11], such as those with inflammatory bowel disease (IBD) or CeD [12]. Celiac disease is a common chronic enteropathy, and its incidence is rising worldwide [13]. It develops in genetically predisposed individuals after the ingestion of gluten, the principal environmental trigger present in wheat and other cereals. The presence of specific HLA class II heterodimers, i.e., DQ, represents the necessary but insufficient genetic asset for the development of the disease. According to the different reported cohorts, the HLA-DQ2.5 heterodimer (DQA1*0501-DQB1*0201 in cis, DQA1*0505-DQB1*0202 in trans) is present in at least 90% of CeD patients, whereas the other risk heterodimers are HLA-DQ8 (DQA1*0301-DQB1*0302), HLA-DQ2.2 (DQA1^∗^0201-DQB1^∗^0202), or HLA-DQ7.5 (DQA1*0505-DQB1*0301). Thus, about 95% of CeD patients present the DQB1*02 allele, as also recently evaluated by a metanalysis study [14]. Gluten peptides reach the lamina propria, where they are deamidated by tissue transglutaminase 2, loaded onto antigen-presenting cells, and then recognized by T cells. However, even if HLA-DQ2 is present in about 30% of the Caucasian population and gluten is a common component of the Western diet, only 2–5% of these subjects develop the disease, indicating that these factors alone are not sufficient. In addition to other genetic components, several other environmental etiological factors have been proposed, such as viral infections [15], impaired commensal homeostasis [16], and increasing gastrointestinal permeability [17].

In this paper, we demonstrate that mNPs (Au-, Ag-, ZnO-, and TiO2-NPs) interact with gliadin and affect the intestinal barrier homeostasis in an in vitro system; in addition, this combination activates both the innate and adaptive immune response in duodenal biopsies of celiac disease patients, but not in controls. Due to their ability to interact with the intestinal mucosa, the introduction of mNPs into the human diet may thus represent one of the environmental factors increasing the percentage of genetically susceptible individuals developing this disease.

## 2. Results

### 2.1. Food Nanoparticles Interact with Digested PT-Gliadin

**Figure 1:**
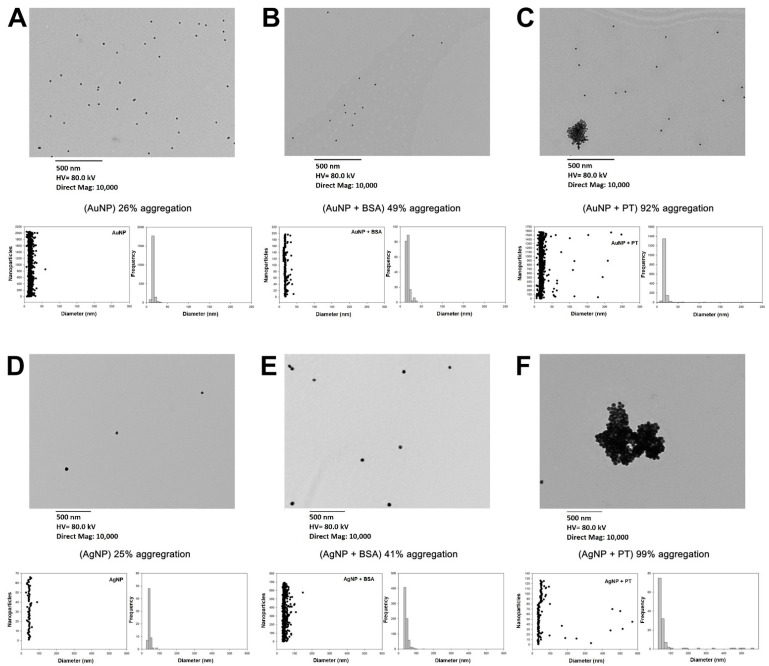
1 Nanoparticles TEM pictures, scatter plots, and histograms of frequency based on the mNPs’ diameter. (**A**) AuNPs; (**B**) AuNPs plus BSA; (**C**) AuNPs plus PT; (**D**) AgNPs; (**E**) AgNPs plus BSA; (**F**) AgNPs plus PT.

To evaluate the possible interactions between mNPs and gliadin, we initially incubated mNPs with or without pepsin-trypsin-digested gliadin (PT) or bovine serum albumin (BSA, known for its interaction with several NPs) and analyzed them by transmission electron microscopy (TEM). As expected, the measured mean particle diameter was 15.37 ± 3.02 nm and 44.36 ± 8.06 nm for Au- and Ag-NPs, respectively. The micrographs showed well-distributed single NPs with a basic rate of aggregation at about 25% when alone (Figure 1A,D). We observed high rates of aggregation, i.e., >90%, after 30 min of incubation for both Au- and Ag-NPs with PT (92% and 99%, respectively, Figure 1C,F), but not with BSA (aggregation rate 49% for AuNPs and 41% for AgNPs, Figure 1 B,E), with the formation of large aggregates with diameters up to 248 and 569 nm, respectively (Figure 1). We did not proceed with the analyses of TiO2- and ZnO-NPs by TEM since they appeared to be poorly distributed and in a cluster, even on their own (data not shown).

**Figure 2:**
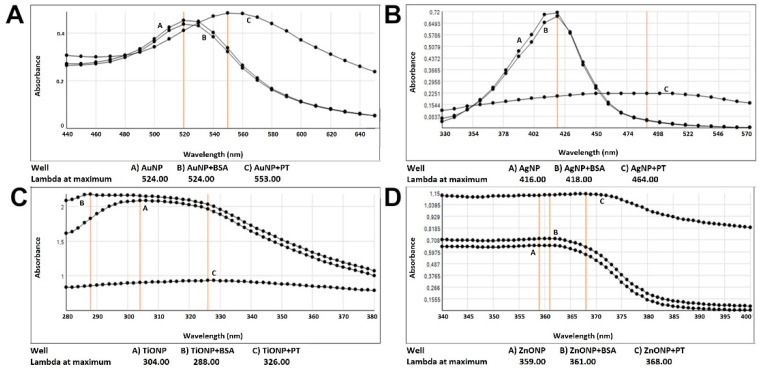
UV–Vis spectra of mNPs. The graphs report the absorbance on the ordinate and the wavelength on the abscissa. (**A**) AuNPs spectra; (**B**) AgNPs spectra; (**C**) TiO2NPs spectra; (**D**) ZnONPs spectra.

We further evaluated the PT–mNPs interaction using surface plasmon resonance (SPR), since mNPs’ characteristic maximum absorbance changes depending on the size, shape, and interaction with proteins (protein corona) [18]. Au- and Ag-NPs spectra presented a well-defined absorption peak (suggesting mNPs in a well-dispersed state), while ZnO- and TiO2-NPs spectra did not. We observed a shift in the SPR after 30 min incubation with BSA with all mNP except for AuNPs (Figure 2), whereas a stronger interaction was detected between all the mNPs and PT, as indicated by the redshift of 48, 29, 9, and 22 nm obtained after 30 min incubation with Ag-, Au-, ZnO-, and TiO2-NPs, respectively. The strong shift, additional maximal absorbance, and broadening of the curves were also consistent with NPs aggregation. The main food source of TiO2NPs is the food-coloring agent E171 [19], which is constituted by different crystalline forms. The UV–Vis spectra of the commercial E171 (Figure S1A) showed a different peak compared to that of TiO2NPs (Figure 2C), suggesting a different composition; although, a wide peak was observed for E171 alone, the broadening of the curve indicating the tendency to aggregate of E171 when combined with PT. To further confirm that the interaction involved gliadin peptides and not the inactivated residual trypsin and pepsin present in the preparation, we also employed PT filtered with a 10 kDa cut-off membrane (able to separate the peptides from the enzymes), obtaining similar results (data not shown).

### 2.2. mNPs +/− PT Affect Cellular Viability

**Figure 3:**
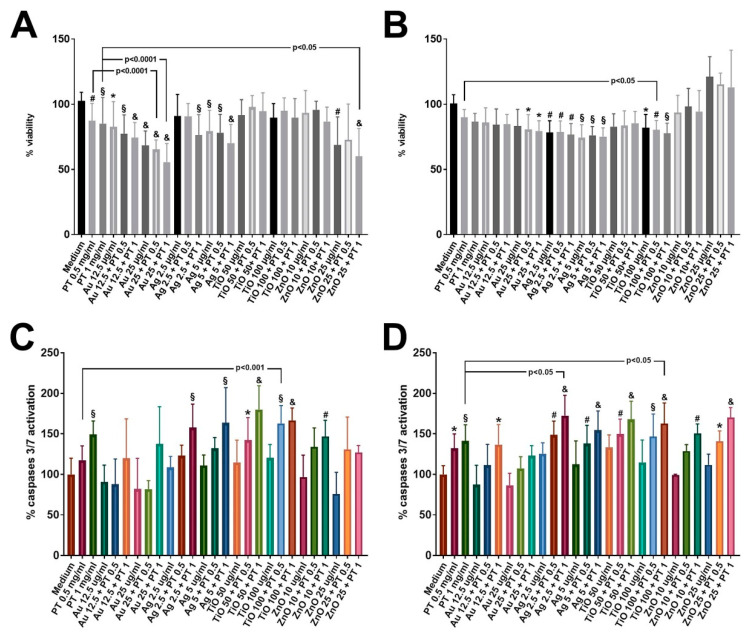
MTT and Caspase 3/7 activation assays. Data are expressed as percentage of untreated cells (Medium). Significance versus untreated cells is indicated above each column (* *p* < 0.05; # *p* < 0.01; § *p* < 0.001; & *p* < 0.0001), whereas versus PT is represented by bars. (**A**) MTT on undifferentiated cells; (**B**) MTT on post-confluent cells; (**C**) apoptosis assay on undifferentiated cells; (**D**) apoptosis assay on post-confluent cells. Data are shown as mean value and SD (*n* = 8).

To assess whether the physical interaction between the single mNPs and the digested gliadin was affecting cellular viability, an MTT assay was initially performed to generate a dose–response curve for the various mNPs on undifferentiated Caco-2 cells (Figure S2A) at several time-points (data not shown). Thus, for further experiments, 24 h was chosen as incubation time, and only mNPs concentrations that did not induce more than 40% of mitochondrial dysfunction (AuNPs: 12.5–25 μg/mL; AgNPs: 2.5–5 μg/mL; TiO2NPs: 50–100 μg/mL; ZnONPs: 10–25 μg/mL) were used, in order to assess the possible additive effect of PT (0.5–1 mg/mL). Since a different effect of mNPs based on cell differentiation had been demonstrated [20], an MTT assay was performed on both undifferentiated (80–90% confluent) and post-confluent Caco-2 cells. In the undifferentiated cells, a significant reduction in cell viability was observed after stimulations with the higher concentrations of either PT-gliadin or Au-, Ag- (at least *p* < 0.001 vs. medium), and ZnO-NPs alone (*p* < 0.01 vs. medium), but worse effects were detected when these mNPs were combined with gliadin (*p* < 0.0001 vs. medium) (Figure 3A). However, only the combination of PT with Au- and ZnO-NPs, but not with AgNPs, significantly reduced viability compared to PT alone (Figure 3A). When post-confluent cells were analyzed, a significant reduction in the cell viability was observed after Ag- and TiO2-NP treatment either alone or combined with PT, as well as after AuNPs combined with PT (*p* < 0.05 vs. medium) (Figure 3B). In addition, data showed a worse effect of these combinations versus the PT alone: TiO2NP treatments in combination with PT were significantly more toxic (*p* < 0.05 vs. PT alone) and the combination of AgNPs plus PT induced a reduction in cell viability with borderline significance (*p* = 0.069 vs. PT alone) (Figure 3B). Moreover, the combination of AuNPs plus PT induced a 10% reduction in cell viability compared to PT alone, although this difference did not reach significance (80.86 ± 4.22 in the combination vs. 90 ± 1.9 in PT, mean ± SEM) (Figure 3B). A similar increase in toxicity was also observed when post-confluent cells were exposed to E171; this food additive, either alone or combined with gliadin, reduced viability compared to unstimulated cells. In addition, the treatment with the combination of E171 and PT 0.5 mg/mL was significantly more damaging compared to PT alone (*p* < 0.05) (Figure S1C). Non-cellular tests were also run, with null or negligible signals (data not shown).

Caspase activation is a transient event that occurs before cellular membrane permeability alterations; therefore, we first performed a time-course analysis employing a cytotoxicity assay able to detect membrane damage. An increase in cytotoxicity became evident at 8 h after stimulations (Figure S2B); thus, we assessed apoptosis activation at an earlier time point (6 h). We observed a significant increase in the caspase 3/7 activation in both undifferentiated and post-confluent Caco-2 cells after stimulations with PT at the higher concentration (*p* < 0.001 vs. medium), with a more robust activation when gliadin was combined with Ag- and TiO2-NPs (*p* < 0.0001 vs. medium) (Figure 3C,D). Indeed, the combination of PT with Ag- or TiO2-NPs resulted in a significantly higher caspase activation compared to PT alone (Figure 3C,D). Even in this case, treatment with E171 induced similar results, with significantly higher levels of caspase activity after PT+E171 treatment vs. PT (Figure S1D,E).

Collectively, the obtained data suggest that food mNPs increase the toxicity exerted by PT on both undifferentiated and post-confluent Caco-2 cells.

### 2.3. Gastrointestinal Barrier Impairment

**Figure 4:**
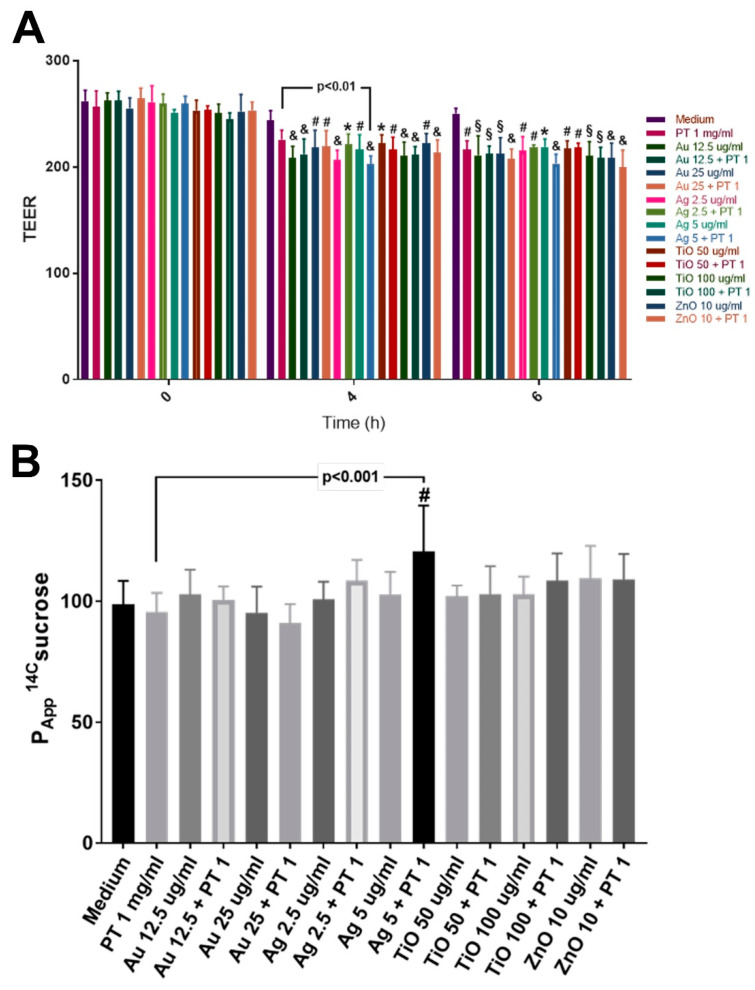
Intestinal barrier impairment. Significance versus untreated cells is indicated above each column (* *p* < 0.05; # *p* < 0.01; § *p* < 0.001; & *p* < 0.0001), whereas versus PT is represented by bars. (**A**) TEER (Ωcm^2^) measured after 0, 4, and 6 h of stimulations; (**B**) apparent paracellular permeability of radiolabeled 14C-sucrose after 24 h of treatments. The values are expressed in cm/min. Data are shown as mean value and SD.

In the development of celiac disease, an important role could be played by intestinal barrier dysfunction. To assess the possible effects of mNPs, Caco-2 cells were seeded on Transwell filters until their differentiation (21 days), and monolayer integrity was assessed by measuring both the transepithelial electrical resistance (TEER) and the passage of 14C-sucrose and 3H-propranolol, probes for the paracellular and transcellular pathways, respectively. Data showed a significant TEER reduction (*p* < 0.05 vs. medium) after 4 and 6 h of stimulation with PT and all the mNPs, either alone or in combination compared to the untreated cells. However, only the combination of PT with AgNPs, at 4 h, was able to significantly reduce TEER in comparison to PT treatment (Figure 4A). In addition, the combination of AgNPs with PT induced a significant increase in the paracellular permeability compared to both the untreated cells and the PT alone (*p* < 0.01 vs. medium; *p* < 0.001 vs. PT) (Figure 4B).

**Figure 5:**
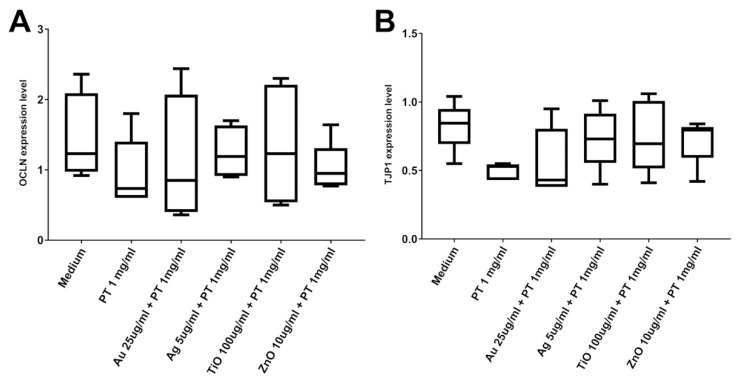
**Tight Junction components** mRNA expression in post-confluent Caco2 cells. (**A**) Occludin (OCLN gene) mRNA expression. (**B**) Zonula Occludens-1 (TJP1 gene) mRNA expression. Box plots represent median, 25th, and 75th percentiles. Whiskers indicate 5th and 95th percentiles.

**Figure 6:**
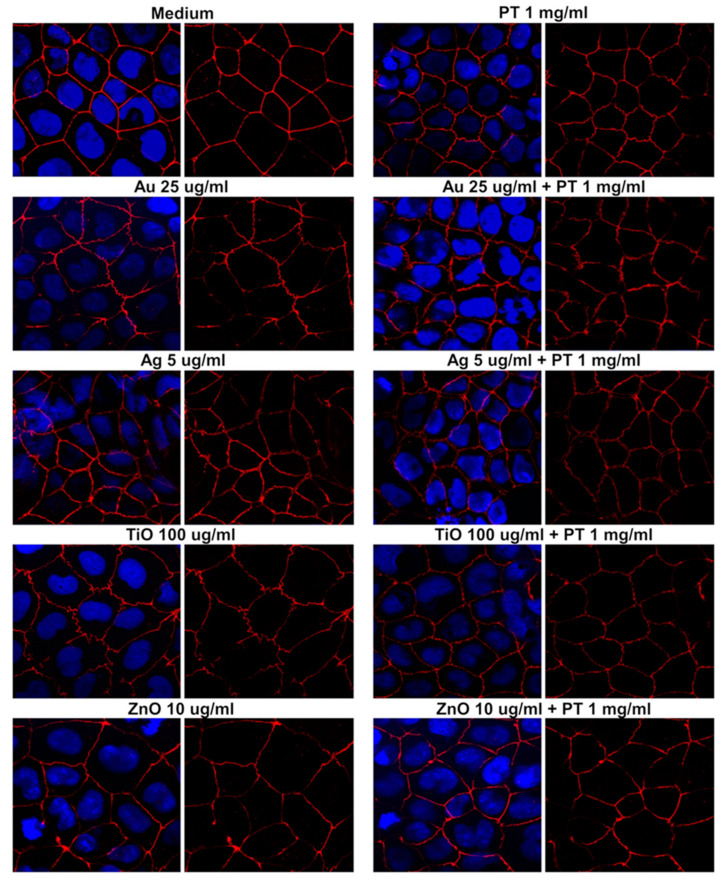
Zonula occludens-1 immunofluorescence in post-confluent Caco2 cells. Left panels show the overlay with 4′,6-diamidino-2-phenylindole (DAPI), and right panels show the ZO-1 signal alone (63× magnification).

**Figure 7:**
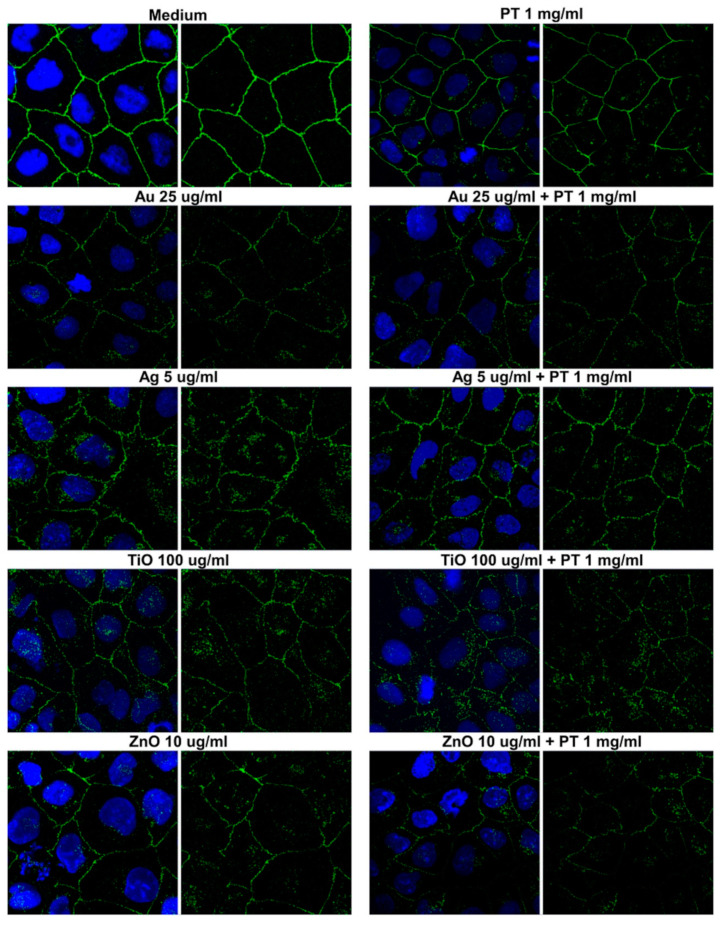
Occludin immunofluorescence in post-confluent Caco2 cells. Left panels show the overlay with DAPI, and right panels show the OCLN signal alone (63× magnification).

To assess if tight junction alterations could be an effect of the various treatments, we evaluated the mRNA level of occludin (OCLN) and zonula occludens-1 (ZO-1) in post-confluent Caco-2 cells. Cells were treated for 24 h with the higher concentration of mNPs with or without PT, but no alteration was detected at the mRNA level (Figure 5). In order to evaluate possible protein expression variation and/or redistribution, we also performed immunofluorescence studies. In controls, the signal of both ZO-1 (Figure 6) and occludin (Figure 7) was localized at the periphery of the cells, delineating the cell contour. Treatment with PT, NPs, or their combination caused a membrane ruffling that was particularly evident for ZO-1, whereas the alteration in the expression/distribution was more pronounced for occludin since a strong decrease in the cell contour signal was observed after incubation with all the different stimuli. In particular, we observed the presence of a punctate intracellular signal after the treatment with Ag-, TiO2-, and ZnONPs, either alone or in combination, suggestive of an alteration of occludin intracellular trafficking. Lastly, we assessed the capacity of E171 to induce OCLN and ZO-1 protein rearrangements, detecting even worse effects after gliadin combination with E171 than with TiO2NPs. In fact, the data obtained suggest a disintegration of the occludin junctions after E171 plus gliadin treatment, an effect confirmed by the ZO-1 signal showing an initial separation of the cells (Figure S1F).

### 2.4. NPs Induce an Immune Response in the Duodenal Mucosa of CeD Patients

We also evaluated the potential effect of the interaction between mNPs and gliadin ex vivo on duodenal biopsies from both celiac (on a gluten-free diet) and healthy subjects.

The activation of the apoptosis pathway was assessed, evaluating the ratio of the mRNA expression of BCL-2 (anti-apoptotic) and BAX (pro-apoptotic) genes. Among the various treatments, we observed a reduction, although not statistically significant, of the BCL-2/BAX ratio only after stimulations with PT in combination with Ag-NPs in biopsies from CeD on a gluten-free diet (GFD) (PT + AgNPs 0.18 ± 0.07 vs. 0.39 ± 0.24 in medium-incubated biopsies) (data not shown). We also investigated the expression of ZO-1 and OCLN genes and, as expected, no alterations of their mRNA levels were induced by 4-hour treatments (data not shown).

**Figure 8:**
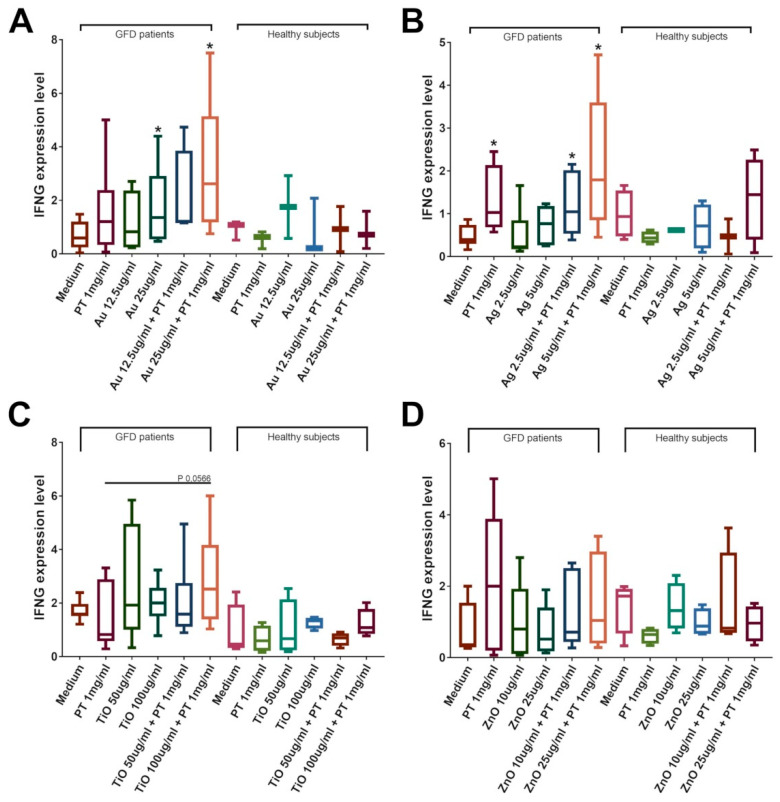
IFNG expression in CeD patients on a GFD and healthy controls. Significance versus untreated biopsies is indicated above each column (* *p* < 0.05), whereas versus PT is represented by bars. Duodenal biopsies stimulated 4 h with PT 1 mg/mL ± (**A**) AuNPs 12.5/25 μg/mL (GFD *n* = 6; Healthy *n* = 3); (**B**) AgNPs 2.5/5 μg/mL (GFD *n* = 6; Healthy *n* = 4); (**C**) TiO2NPs 50/100 μg/mL (GFD *n* = 7; Healthy *n* = 4); (**D**) ZnONPs 10/25 μg/mL (GFD *n* = 5; Healthy *n* = 4). Box plots represent median, 25th, and 75th percentiles. Whiskers indicate 5th and 95th percentiles.

**Figure 9:**
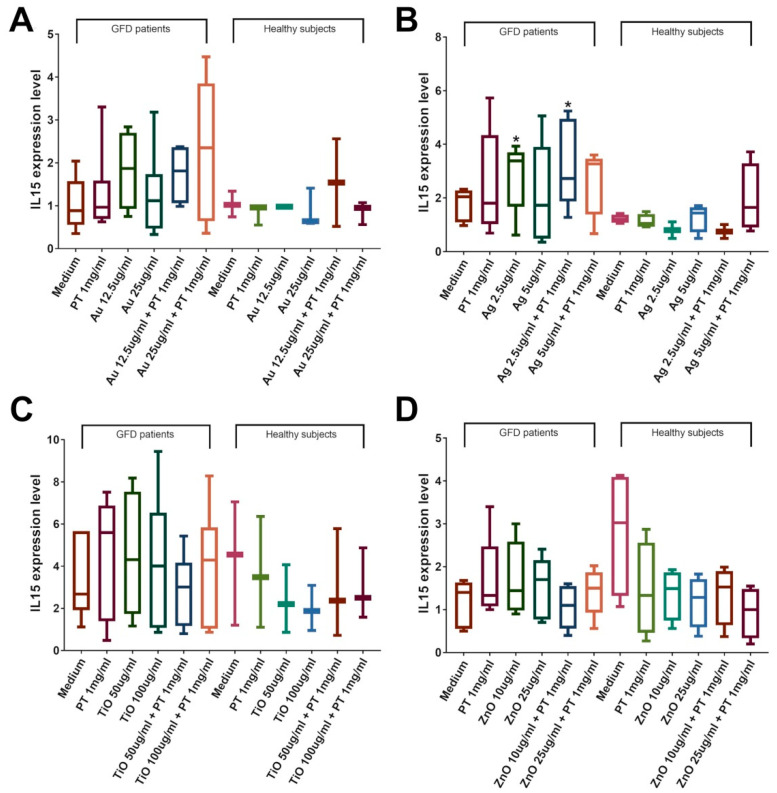
IL-15 expression in CeD patients on a GFD and healthy controls. Significance versus untreated biopsies is indicated above each column (* *p* < 0.05), whereas versus PT is represented by bars. Duodenal biopsies stimulated 4 h with PT 1 mg/mL ± (**A**) AuNPs 12.5/25 μg/mL (GFD *n* = 6; Healthy *n* = 3); (**B**) AgNPs 2.5/5 μg/mL (GFD *n* = 6; Healthy *n* = 4); (**C**) TiO2NPs 50/100 μg/mL (GFD *n* = 7; Healthy *n* = 4); (**D**) ZnONPs 10/25 μg/mL (GFD *n* = 5; Healthy *n* = 4). Box plots represent median, 25th, and 75th percentiles. Whiskers indicate 5th and 95th percentiles.

**Figure 10:**
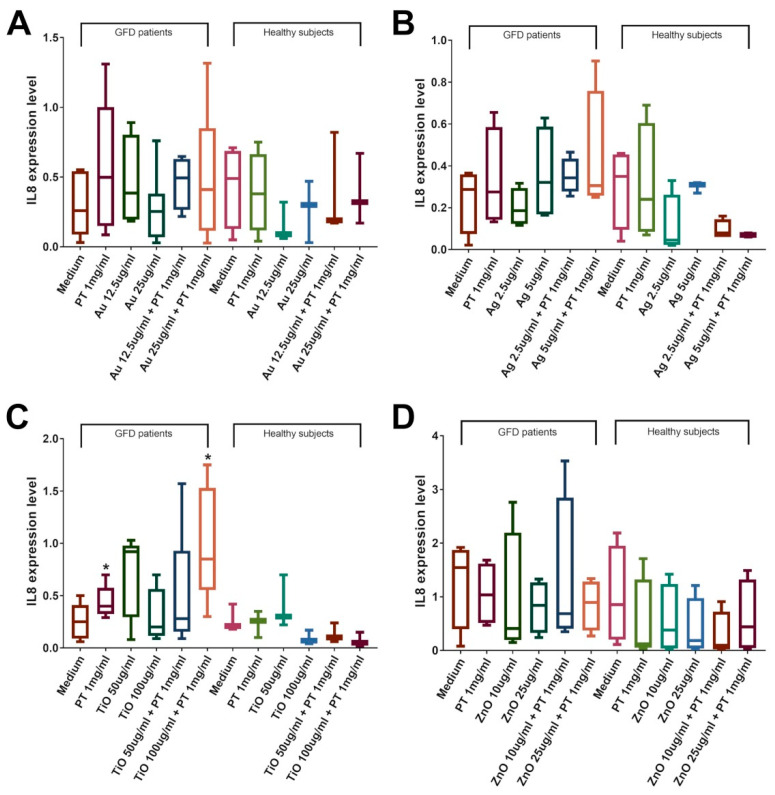
IL-8 expression in CeD patients on a GFD and healthy controls. Significance versus untreated biopsies is indicated above each column (* *p* < 0.05), whereas versus PT is represented by bars. Duodenal biopsies stimulated 4 h with PT 1 mg/mL ± (**A**) AuNPs 12.5/25 μg/mL (GFD *n* = 6; Healthy *n* = 3); (**B**) AgNPs 2.5/5 μg/mL (GFD *n* = 6; Healthy *n* = 4); (**C**) TiO2NPs 50/100 μg/mL (GFD *n* = 7; Healthy *n* = 4); (**D**) ZnONPs 10/25 μg/mL (GFD *n* = 5; Healthy *n* = 4). Box plots represent median, 25th, and 75th percentiles. Whiskers indicate 5th and 95th percentiles.

To assess if mNPs could also modify the immune system response, we evaluated the mRNA levels of the most representative cytokines involved in the development of the celiac disease lesions: IFNɣ for the adaptive immune system and IL-15 and IL-8 for the innate immunity. We observed their increase only in duodenal biopsies from celiac patients on a gluten-free diet but not in those from healthy individuals. Results showed an increase in IFNɣ expression after stimulations with PT. A significant increase in IFNɣ mRNA levels was observed in CeD biopsies after incubation with AuNPs at a concentration of 25 µg/mL, both with and without the addition of PT (*p* < 0.05 vs. control, Figure 8A). Interestingly, the combination of AuNPs plus PT induced a higher IFNɣ expression compared to PT alone, although this difference did not reach significance (3.19 ± 0.84 in the combination vs. 1.59 ± 0.57 in PT, mean ± SEM). Conversely, AgNPs at both concentrations were able to induce a significant increase in INFɣ expression only in combination with PT versus medium (*p* < 0.05 vs. controls, Figure 8B); even, in this case, AgNPs in combination with PT were able to increase IFNɣ mRNA level compared to PT alone (2.16 ± 0.66 in the combination vs. 1.30 ± 0.31 in PT, mean ± SEM). TiO2NPs exposure did cause a modest increase in INFɣ only when it was in combination with PT, whereas ZnO did not have any effect (Figure 8C,D). The analysis of IL-15 expression revealed a significant increase in the mRNA amount of the cytokine after stimulations with AgNPs at the concentration of 2.5 µg/mL ± PT (*p* < 0.05 vs. control, Figure 9B), whereas no other stimulation was able to induce a significant change. No alteration of the IL-8 level was detected after Au-, Ag-, or ZnO-NPs stimulation, whereas a significant increase was observed after treatments with PT and TiO2NPs (*p* < 0.05 vs. control, Figure 10C). Again, the combination induced a higher increase in expression (1.01 ± 0.25 in the combination vs. 0.44 ± 0.07 in PT, mean ± SEM), although the difference did not reach significance. To further evaluate innate immunity, TLR2 and TLR4 mRNA expression were also investigated (Figures S3 and S4). Interestingly, results showed a statistically significant increase of TLR2 after treatment with TiO2NPs + PT (Figure S3C). No alteration of their expression was observed after treatments with the other NPs alone or in combination with the gliadin.

## 3. Discussion

Several studies showed that the mNPs can alter the intestinal microbiota composition, impair the gastrointestinal barrier permeability, and induce immune modulation [21,22], thus having an important effect on patients with diseases characterized by intestinal damage and immune response alterations such as inflammatory bowel diseases [10,12,23]. Herein, we continue in that direction, pointing to CeD as another possible disorder in which the dietary intake of mNPs could play a significant role. Moreover, our work is one of the few testing the toxicity of the mNPs associated with food components [23,24,25,26,27].

We initially evaluated the potential interaction between mNPs and PT by TEM and UV–Vis analyses, showing that gliadin peptides can bind the surface of the mNPs herein studied, inducing their aggregation. This could induce a change in size that can affect mNPs’ performance in crossing biological barriers, the epithelial barrier included [28]. However, it has been shown that the average optimal reported size for NP transcytosis in the gastrointestinal tract varies according to the cell type. In fact, enterocytes preferentially take up NPs of 20–100 nm, whereas particles with 100–600 nm in diameter are transported by M cells [29]. Thus, the aggregates formed by gliadin and the mNPs could cause a change in the type of cells able to vehiculate their passage across the intestinal barrier, in absence of any other damage able to increase the paracellular transit. The passage through M cells, however, could play an important role in CeD since these cells are specialized in the translocation of large molecules from the intestine to the immune cells. It must also be noted that these data have been obtained in a “simplified” environment, i.e., not evaluating the interactions with other food components or changes in pH, which actually occur in the gastrointestinal tract [6]. The interaction between NPs and food and/or digestion juices have been analyzed by several papers, but the published data do not seem to clarify these issues, due to the differences in the type of NPs, their size, the chosen food components, or the simulation of the digestive process [30,31,32,33,34].

Even the cytotoxic effect of digested NPs can vary in different studies, as recently reported by Cao et al. [35] and Marucco et al. [36]. In the first paper, the toxicity of digested food-grade TiO2 was observed to be higher for E171 digested without the presence of other food components, but there was no comparison with undigested TiO2. On the other hand, Marucco et al. [36] found no difference in cytotoxicity between digested and undigested TiO2. The cytotoxic effects, however, can also depend on the formation of a protein corona as well as the aggregation state of the NPs, as observed by Wang et al. [27] and Albanese et al. [37]. The propensity of NPs to be dissolved and to release heavy metals is also a key point to be considered in the study of the NPs’ toxicity. Recently, it has been shown that copper, silver, and titania nanoparticles can release about 8 μg/mL of ions when dissolved in oxygen-saturated aqueous suspension, starting from 48 h. These levels are low compared to those found in natural media, thus suggesting that the toxicity due to released ions in our experimental conditions should be negligible [38]. In our model, mNPs alone were able to induce toxicity in Caco-2 cells, although the effects were quite different according to the cell status (i.e., proliferating or post-confluent), in particular for Au- and TiO2-NPs. Moreover, stimulations with Au- and ZnO-NPs combined with PT induced a significant reduction of cell viability compared to PT alone in non-confluent cells, whereas TiO2NPs induced the same effect in the post-confluent ones. Apoptosis is a pivotal mechanism involved in CeD pathogenesis [39], and in Caco-2 cells, PT alone induced significant activation of apoptosis, as demonstrated in other works [39]. However, the activation of apoptosis after stimulations with TiO2- and Ag-NPs combined with PT was significantly higher compared to the cells treated with PT alone. These results suggest a possible additive effect of mNPs and PT that also depends on the cell characteristics, as previously detected by Hanley et al. [5], who showed a different NP toxicity based on the cell proliferative status. This could be important not only in considering the normal mucosa but also in the case of celiac disease, since the exposure of duodenal mucosa to gluten leads to a situation of hyperproliferation in an attempt to restore the normal intestinal architecture, thus changing the ratio of completely differentiated/immature cells.

From the literature, it is clear that both gliadin and mNPs can impair the gastrointestinal barrier [17,21,40,41]. Since this alteration may facilitate the gliadin passage from the intestinal lumen to the lamina propria and, in turn, increase the immune response, we evaluated whether the combination of PT-mNPs could cause worse effects than the single components. TEER values were significantly decreased starting from 4 h after treatments, but an additional effect was only observed after the stimulation of AgNPs + PT versus PT alone. In addition, when paracellular permeability was analyzed, a significant increase in the passage of radiolabeled sucrose was observed after the same stimulation versus both the untreated cells and PT alone. The data obtained on paracellular permeability differ from what was reported by Sander et al. [40], in which an increase in the paracellular transport of the 4 kDa FITC dextran marker was registered after stimulation with the digested gliadin alone. However, although the same cellular model was employed, the timing of the experiment and the permeability marker used by the authors were different, a fact that could explain the difference in the obtained results. TJ protein redistribution was observed by immunofluorescence, with both OCLN and ZO1 showing membrane ruffling after all the stimulations. This pattern was slightly present after stimulation with the PT alone, which is in line with the findings of Sander et al. [40], but the combination of the NP treatment with PT increased the disruption of cell-to-cell junctions. Moreover, we observed an increase of cytosolic punctate staining consistent with an alteration of the intracellular vesicular trafficking of the occludin protein, particularly after treatment with Ag- and TiO2-NPs, both alone and combined with PT.

TiO2NPs are commonly used as a food-whitening agent in E171, but their size, composition, and concentration are often controversial. The composition of E171 involves a mixture of anatase (usually the most elevated component) and rutile with P, Si, and Al contaminants, and it is estimated that its NPs components range from 10% to 45%, according to the commercial source [19,42]. Therefore, we also performed experiments with the commercial food-grade E171 to confirm our findings on TiO2NPs. Although UV–Vis spectra proved a different composition between the commercial E171 and TiO2NPs, both cytotoxic effects and TJ protein rearrangements after E171 exposure were comparable to those induced by TiO2NPs. These results support the need for a profound rethinking of the guidelines for the use of this additive in commercially available food. The FDA allows E171 at up to 1% of the weight of the food, whereas in Europe, this food additive is authorized ad quantum satis [43]. The percentage of its absorption, as well as the entry route, remains to be completely elucidated, although a recent paper by Comera et al. [44] demonstrated that E171 absorption in mice occurs through the paracellular route. The authors were also able to demonstrate the presence of E171 in the lamina propria, a fact that could be important since the binding of gluten peptides to mNPs may lead to an increased passage of immunogenic molecules able to trigger the immune response (hypothesis of the “Trojan horse”) [11].

In addition, metal nanoparticles can potentiate the immune response [45], which represents a potential benefit in biomedical application, but could be harmful for dietary mNPs. Various studies proved that food particles, particularly E171, could have a role in IBDs [10,12,23,46]; Ruiz et al. [23] performed the studies on DSS-induced ulcerative colitis mice, and Powell et al. [12] and Evans et al. [10] on biopsy specimens from IBD patients, showing an increased activation of the innate immune system after E171 exposure.

Although our results demonstrate the deleterious effects of the interaction of mNPs and gliadin peptides on an in vitro model system, further studies are necessary to confirm these effects in the complex environment of the gastrointestinal tract. In fact, in addition to the variables cited above, i.e., the presence of other food components and of intestinal juices, other factors could modify the interaction between NPs and the epithelium. Particularly, in addition to the epithelium, the gastrointestinal barrier also includes the mucus layer, which can trap the mNPs, reducing their uptake and toxic effects. In turn, mNPs can alter the mucus layer, affecting its thickness or its composition [47,48].

Thus, to evaluate the effect of mNPs +/− gliadin in a system more comparable to the intestine, we employed an ex vivo system with duodenal biopsies obtained from CeD patients and healthy subjects. For this study, we selected CeD on a gluten-free diet, with absent or minimal residual inflammation and normal villi/crypt ratio; this choice allowed us to better evaluate the response to the stimuli in term of cytokine production, and also to have a number of enterocytes comparable with that present in control biopsies. Cytokines and TLRs were selected as representative of the involvement of the immune system in CeD; IFNɣ was evaluated for the adaptive response; IL-15, IL-8, TLR2, and TLR4 were evaluated for the innate response. Interestingly, an increase was observed only after stimulations of the duodenal biopsies from CeD patients, but not from controls, suggesting that although mNPs could be innocuous for healthy subjects, they could be harmful for the celiac subjects. Although a recent paper by Gokulan et al. [49] analyzes the effect of AgNPs on the human intestinal mucosa, it is quite difficult to compare their data with ours, due to differences in the time of the exposure as well as in the AgNPs concentration. However, even in their experimental conditions, no significant increase in IFNɣ expression was observed after 2 h exposure in normal mucosa. Moreover, the higher increases in expression in CeD biopsies were induced by stimulation with the combination of mNPs and PT, although the difference did not reach significance compared to PT alone.

The effect was not the same for all the tested mNPs since AuNPs induced IFNɣ expression, Ag-NP treatments induced IL-15 and IFNɣ expression, whereas TiO2NPs increased IL-8 and TLR2, but also IFNɣ levels. In this latter case, the increase observed after the combined treatment reached a borderline significance compared to PT alone. The IFNɣ increase, directly linked to the adaptive immune response, leads to the hypothesis that the analyzed mNPs may interfere with the antigenic presentation of gliadin mediated by dendritic cells. In fact, Schanen et al. [50] demonstrated that TiO2 nanoparticles induced dendritic cell maturation and naïve T cell activation and proliferation; Fogli et al. [3] showed how different NP cores and coatings can induce different responses in dendritic cells, and Galbiati et al. [51] proved an immunostimulatory effect on THP-1 cells and peripheral blood monocytes. Interestingly, data obtained in a mouse model have suggested that the prolonged ingestion of TiO2NPs is able to alter the Th1/Th2 balance in the intestine, favoring the Th2 response, an imbalance that, in turn, can lead to epithelial damage. In fact, in this model, the authors observed a decrease in the villi/crypts ratio, with histology similar to findings in CeD [52]. Although the immune response in CeD is mainly Th1, an increase in the secretion of the Th2 cytokine IL-13 has been reported in refractory CeD, and this mechanism could be important in maintaining the villous damage [53]. Therefore, studies regarding dendritic and T cell activation/proliferation with the combination of mNPs and PT are needed to clarify whether mNPs only promote the gliadin passage into the lamina propria or whether they can also increase the immune cell response to gliadin. Finally, there is another possible interaction between mNPs and intestinal components that was not the focus of this paper but deserves further investigation, i.e., the effect of mNPs on microbiota [48]. In fact, the interaction works both ways since microbiota can metabolize mNPs, changing their biological effects, whereas mNPs can affect the microbiota composition. Alteration of microbiota could be quite important in an autoimmune disorder such as celiac disease, due to the possible interactions between microbiota and immune system, as well as microbiota and gluten. However, it must be underlined that a specific microbiota signature has not been identified yet, neither in children [54] nor adults [55]. In addition, most of the published studies have been cross-sectional and thus unable to discriminate the causal role of an altered microbiota composition; therefore, prospective studies are needed, in particular evaluating the duodenal microbiota.

To the best of our knowledge, our work appears to be the first to document the synergy between food mNPs and gliadin peptides, leading to the hypothesis that mNPs could be one of the unknown factors playing a role in the increasing CeD incidence. The specific mechanisms involved, and whether the mNPs lead to a higher gliadin passage into the lamina propria [7,56] or increase the immune cell responsiveness to the gliadin [57], need to be further evaluated.

## 4. Materials and Methods

### 4.1. Peptic-Tryptic Digested Gliadin (PT)

The digestion procedure was performed as described by Frazer et al. [58], with some modifications. Briefly, gliadin (Sigma-Aldrich, St Louis, MO, USA) was digested with pepsin (0.1 M HCl, pH 1.8) (Sigma-Aldrich, St Louis, MO, USA) and then trypsin (pH 7.8) (Sigma-Aldrich, St Louis, MO, USA) at 37 °C for 4 h with vigorous agitation. Adjustment of the pH to 4.5 resulted in a precipitate, which was removed by centrifugation. To inhibit the residual enzymatic activity, both N-tosyl-l-phenylalanine chloromethyl ketone and N- α-tosyl-l-lysine chloromethyl ketone hydrochloride (Sigma-Aldrich, St Louis, MO, USA) were used. After dialysis, PT was sterile filtered and lyophilized. The resulting powder was dissolved in sterile water and stored at −20° C.

### 4.2. Transmission Electronic Microscope (TEM) and UV-Vis Spectra Analyses

AgNPs (40 nm in diameter), TiO2NPs (<25 nm in diameter, anatase), and ZnONPs (<100 nm in diameter) were purchased from Sigma-Aldrich (St Louis, MO, USA). AuNPs (15 nm in diameter) were obtained from Cytodiagnostics Inc. (Burlington, Canada). E171 whitening agent was purchased from an Italian commercial supplier of food coloring. For TEM analyses, Ag-, Au-, TiO2-, and ZnO-NPs were sonicated for 1 min then incubated with/without PT or bovine serum albumin (BSA) (Sigma-Aldrich, St Louis, MO, USA) for 30 min. A drop of these suspensions was deposited onto carbon-coated copper grids and allowed to dry at room temperature. Images were obtained using Hitachi H-7500 TEM (Hitachi Ltd., Tokyo, Japan) and analyzed using ImageJ^TM^ software version 1.48 (NIH, Bethesda, MD, USA). The surface area of mNPs/aggregate was used to calculate the radius, diameter, and three-dimensional area. The number of mNPs in every cluster was obtained, dividing these values for the three-dimensional area of one mNPs, considering 15 nm as AuNP’s diameter and 40 nm as AgNP’s diameter (as the datasheet specifics). All the clusters with two or more NPs were regarded as aggregates. UV–Vis spectra were acquired, using 2-nanometer intervals after 30 min of mNPs incubation with/without PT or BSA with a SpectraMax Plus 384 Microplate Reader (Molecular Devices, San Jose, CA, USA). For each condition, three separate experiments were performed, and five images for each experiment were analyzed.

### 4.3. Cell Viability Assays

Caco-2 cells (Istituto Sieroterapico, Bergamo, Italy) were cultured in complete DMEM supplemented with 100 IU/mL penicillin/2 mM L-Glutamine and 10% fetal bovine serum at 37 °C (Euroclone, Pero, MI, Italy) in a 5% CO_2_ atmosphere. For all the viability assays, undifferentiated cells were grown until 80–90% confluence, and post-confluent cells were grown until domes were formed, then stimulations were performed. For the MTT assay, after 6/24 h of stimulation, 3-(4.5-dimethylthiazolo-2-yl)-2.5-diphenytetrasolium bromide (Sigma-Aldrich, St Louis, MO, USA) was added at a concentration of 0.5 mg/mL and incubated for 2 h at 37 °C in a 5% CO_2_ atmosphere. The formed salts were dissolved in 100% EtOH, and the plates were read at 570 nm with a Model 550 Microplate Reader (Bio-Rad,Hercules, CA, USA). Eight separate experiments were performed for each condition. The CellTox™ Green Cytotoxicity Assay (Promega, Milan, Italy) was used to determine the appropriate time for measuring the transient caspase activity. For the Caspase-Glo^®^ 3/7 assay (Promega, Milan, Italy), cells were treated for just 6 h, the luminogenic substrate was added, and plates incubated at room temperature for 2 h and read with the Tecan Infinite^®^ 200 PRO plate reader (Mannendorf, Switzerland).

### 4.4. Transepithelial Electrical Resistance (TEER) and Apparent Permeability (Papp)

Caco-2 cells were seeded on Transwell polyester inserts (0.4 µm pore size) with a density of 165 × 103 cells/insert. TEER was monitored with STX2 electrode Epithelial Volt-Ohm (TEER) Meter (World Precision Instruments, Sarasota, FL). Only inserts with TEER ≥ 300 Ω/cm^2^ were used. Paracellular Papp was assessed using [14C]-sucrose and the transcellular Papp by [3H]-propranolol transport (0.045 mCi/upper chamber for each probe). After 150 min, Papp was determined as

Papp = *dQ*/*dt*·1/*C*0*A*

where *dQ*/*dt* is the transport of the probes from the upper to the lower chamber as a function of time, *C*0 is the initial probe concentration, and A is the superficial area of each insert. For each of the conditions, three separate experiments were performed.

### 4.5. Immunofluorescence

Caco-2 cells were plated on 35-millimeter, collagen-coated glass-bottom dishes and were treated at 4 days post-confluence. After 24 h of stimulation, cells were fixed in methanol for 10 min at −20 °C, washed three times with high salt buffer, and incubated overnight at 4 °C with Anti-ZO1 (1:100, Cat. 402200; Invitrogen, Thermo Fisher Scientific, Waltham, MA, USA) or Anti-OCLN (1:100, Cat. 331588, Invitrogen, Thermo Fisher Scientific, Waltham, MA, USA) primary antibodies in BSA 1X or BSA 1X with 0.1% saponin, respectively. For Anti-ZO1 Ab, cells were washed and treated with the secondary antibody Alexa Fluor® 568 Goat Anti-Rabbit (IgG) (1:100; Abcam, Cambridge, UK) at room temperature for 1 h. One micromolar of DAPI(Sigma-Aldrich, St Louis, MO, USA) was used to stain cell nuclei. Images were acquired by an SM710 inverted confocal laser scanning microscope (ZEISS, Oberkochen, Germany).

### 4.6. Patients’ Biopsies

Biopsy specimens were collected by upper endoscopy from 15 healthy subjects (undergoing the exam for gastro-esophageal reflux and/or dyspepsia, H. pylori negative) and 26 celiac patients on gluten-free diets for at least 1 year, with absent or minimal inflammatory infiltrate (Marsh 0–1) to better evaluate the cytokine production. Biopsies were treated in vitro with mNPs and/or PT for 4 h at 37 °C and at 5% CO_2_, frozen in liquid nitrogen and maintained at −80 °C until RNA extraction. The study was approved by the ethics committee of the Fondazione IRCCS Ca’ Granda Ospedale Maggiore Policlinico (protocol number 167/2012), Milan, Italy, and informed consent was obtained from all patients.

### 4.7. RNA Extraction and RT-qPCR

Total RNA was extracted using the miRCURY RNA Isolation Kit (Exiqon, Vedbaek, Denmark) following the manufacturer’s instructions. RNA quality analysis and quantification were performed by NanoDrop 1000 Spectrophotometer (Applied Biosystems, Thermo Fisher Scientific, Waltham, MA, USA). High Capacity cDNA Reverse Transcription kit (Applied Biosystems, Thermo Fisher Scientific, Waltham, MA, USA) was used with random primers to obtain cDNAs, and TaqMan™ Gene Expression Assays (Applied Biosystems, Thermo Fisher Scientific, Waltham, MA, USA) were used for gene expression studies. Then, qPCR was performed using a 7900HT Fast Real-Time PCR System (Applied Biosystems, Thermo Fisher Scientific, Waltham, MA, USA). For all analyses, each sample was examined in triplicate. The used probes are: OCL hs00170162_m1; TJP1 hs01551861_m1; IFNG hs00989291_m1; IL15 hs01003716_m1; IL8 hs00174103_m1; HPRT1 hs99999909_m1; BAX hs00180269_m1; BCL2 hs00608023_m1; TLR2 hs00152932_m1; TLR4 hs01060206_m1.

All data were normalized using HPRT1 (Hypoxanthine Phosphoribosyltransferase 1), and the relative expression was assessed by the 2^-ΔΔCt^ method using an external control.

### 4.8. Statistical Analyses

Data obtained by experiments with different stimulations on cells were compared with ANOVA multiple comparisons, both against the basal condition and the PT stimulation. ANOVA on ranks followed by Dunn’s posthoc test was used when data failed the equal variance test. Experiments were conducted with at least *n* = 3 and in triplicate. A paired *t*-test was used to compare the gene expression data of ex vivo stimulations. Evaluation of outliers was performed using the Grubbs’ and ROUT tests. All the statistical evaluation was performed with SYSTAT software (SPSS, Chicago, IL, USA).

## 5. Conclusions

Our work documents the synergy between food mNPs and gliadin peptides in causing damage to an in vitro model of the intestinal barrier but, more importantly, demonstrates that mNPs plus gliadin are able to trigger an increase in cytokine production in duodenal biopsies from celiac patients but not from controls. These results thus suggest that the effects of mNPs on healthy subjects could be negligible, but they could represent an additional risk factor for celiac patients, being able to elicit an inflammatory response and possibly induce immune activation. In fact, the potential ability of the various mNPs to facilitate the passage of potential immunogenic molecules from the lumen to the lamina propria could increase antigen availability, which, in turn, could lead to the activation of the immune system. Based on our results, studies on the interaction between food additives and CeD should be increased, especially considering the broad spectra of used additives, which, apart from mNPs, also include transglutaminase and gluten nanoparticles [59], in order to assess their role in the increase in individuals developing this pathology among the genetically susceptible ones.

## Data Availability

Data available from the corresponding author upon reasonable request.

## Supplementary Materials

The following are available online at https://www.mdpi.com/article/10.3390/ijms22116102/s1.

## References

[1] Peralta-Videa J.R., Zhao L., Lopez-Moreno M.L., de la Rosa G., Hong J., Gardea-Torresdey J.L. (2011). Nanomaterials and the environment: A review for the biennium 2008–2010. J. Hazard. Mater..

[2] EFSA (2011). Scientific Committee Guidance on the risk assessment of the application of nanoscience and nanotechnologies in the food and feed chain. EFSA J..

[3] Fogli S., Montis C., Paccosi S., Silvano A., Michelucci E., Berti D., Bosi A., Parenti A., Romagnoli P. (2017). Inorganic nanoparticles as potential regulators of immune response in dendritic cells. Nanomedicine.

[4] Sahani S., Sharma Y.C. (2021). Advancements in applications of nanotechnology in global food industry. Food Chem..

[5] Hanley C., Thurber A., Hanna C., Punnoose A., Zhang J., Wingett D.G. (2009). The Influences of Cell Type and ZnO Nanoparticle Size on Immune Cell Cytotoxicity and Cytokine Induction. Nanoscale Res. Lett..

[6] Bellmann S., Carlander D., Fasano A., Momcilovic D., Scimeca J.A., Waldman W.J., Gombau L., Tsytsikova L., Canady R., Pereira D.I.A. (2015). Mammalian gastrointestinal tract parameters modulating the integrity, surface properties, and absorption of food-relevant nanomaterials. Wiley Interdiscip. Rev. Nanomed. Nanobiotechnol..

[7] Lerner A., Matthias T. (2015). Changes in intestinal tight junction permeability associated with industrial food additives explain the rising incidence of autoimmune disease. Autoimmun. Rev..

[8] Medina-Reyes E.I., Rodríguez-Ibarra C., Déciga-Alcaraz A., Díaz-Urbina D., Chirino Y.I., Pedraza-Chaverri J. (2020). Food additives containing nanoparticles induce gastrotoxicity, hepatotoxicity and alterations in animal behavior: The unknown role of oxidative stress. Food Chem. Toxicol.

[9] Vance M.E., Kuiken T., Vejerano E.P., McGinnis S.P., Hochella M.F., Rejeski D., Hull M.S. (2015). Nanotechnology in the real world: Redeveloping the nanomaterial consumer products inventory. Beilstein J. Nanotechnol..

[10] Evans S.M., Ashwood P., Warley A., Berisha F., Thompson R.P., Powell J.J. (2002). The role of dietary microparticles and calcium in apoptosis and interleukin-1β release of intestinal macrophages. Gastroenterology.

[11] Fasano A. (2012). Leaky Gut and Autoimmune Diseases. Clin. Rev. Allergy Immunol..

[12] Powell J., Harvey R., Ashwood P., Wolstencroft R., Gershwin M., Thompson R. (2000). Immune Potentiation of Ultrafine Dietary Particles in Normal Subjects and Patients with Inflammatory Bowel Disease. J. Autoimmun..

[13] King J.A., Jeong J., Underwood F.E., Quan J., Panaccione N., Windsor J.W., Coward S., Debruyn J., Ronksley P.E., Shaheen A.-A. (2020). Incidence of Celiac Disease Is Increasing Over Time: A Systematic Review and Meta-analysis. Am. J. Gastroenterol..

[14] Poddighe D., Rebuffi C., De Silvestri A., Capittini C. (2020). Carrier frequency of HLA-DQB1* 02 allele in patients affected with celiac disease: A systematic review assessing the potential rationale of a targeted allelic genotyping as a first-line screening. World J. Gastroenterol..

[15] Lerner A., Arleevskaya M., Schmiedl A., Matthias T. (2017). Microbes and Viruses Are Bugging the Gut in Celiac Disease. Are They Friends or Foes?. Front. Microbiol..

[16] Caminero A., Galipeau H.J., McCarville J.L., Johnston C.W., Bernier S.P., Russell A.K., Jury J., Herran A.R., Casqueiro J., Tye-Din J.A. (2016). Duodenal Bacteria From Patients With Celiac Disease and Healthy Subjects Distinctly Affect Gluten Breakdown and Immunogenicity. Gastroenterology.

[17] Drago S., El Asmar R., Di Pierro M., Clemente M.G., Sapone A.T.A., Thakar M., Iacono G., Carroccio A., D’Agate C., Not T. (2006). Gliadin, zonulin and gut permeability: Effects on celiac and non-celiac intestinal mucosa and intestinal cell lines. Scand. J. Gastroenterol..

[18] Mirsadeghi S., Dinarvand R., Ghahremani M.H., Hormozi-Nezhad M.R., Mahmoudi Z., Hajipour M.J., Atyabi F., Ghavami M., Mahmoudi M. (2015). Protein corona composition of gold nanoparticles/nanorods affects amyloid beta fibrillation process. Nanoscale.

[19] Yang Y., Doudrick K., Bi X., Hristovski K., Herckes P., Westerhoff P., Kaegi R. (2014). Characterization of Food-Grade Titanium Dioxide: The Presence of Nanosized Particles. Environ. Sci. Technol..

[20] Gerloff K., Pereira D.I., Faria N., Boots A.W., Kolling J., Förster I., Albrecht C., Powell J.J., Schins R.P. (2013). Influence of simulated gastrointestinal conditions on particle-induced cytotoxicity and interleukin-8 regulation in differentiated and undifferentiated Caco-2 cells. Nanotoxicology.

[21] Brun E., Barreau F., Veronesi G., Fayard B., Sorieul S., Chanéac C., Carapito C., Rabilloud T., Mabondzo A., Herlin-Boime N. (2014). Titanium dioxide nanoparticle impact and translocation through ex vivo, in vivo and in vitro gut epithelia. Part. Fibre Toxicol..

[22] Borgognoni C.F., Kim J.H., Zucolotto V., Fuchs H., Riehemann K. (2018). Human macrophage responses to metal-oxide nanoparticles: A review. Artif. Cells Nanomed. Biotechnol..

[23] Ruiz P.A., Morón B., Becker H.M., Lang S., Atrott K., Spalinger M.R., Scharl M., Wojtal K.A., Fischbeck-Terhalle A., Frey-Wagner I. (2017). Titanium dioxide nanoparticles exacerbate DSS-induced colitis: Role of the NLRP3 inflammasome. Gut.

[24] Cao X., Ma C., Gao Z., Zheng J., He L., McClements D.J., Xiao H. (2016). Characterization of the Interactions between Titanium Dioxide Nanoparticles and Polymethoxyflavones Using Surface-Enhanced Raman Spectroscopy. J. Agric. Food Chem..

[25] Fang X., Jiang L., Gong Y., Li J., Liu L., Cao Y. (2017). The presence of oleate stabilized ZnO nanoparticles (NPs) and reduced the toxicity of aged NPs to Caco-2 and HepG2 cells. Chem. Interact..

[26] Martirosyan A., Grintzalis K., Polet M., Laloux L., Schneider Y.-J. (2016). Tuning the inflammatory response to silver nanoparticles via quercetin in Caco-2 (co-)cultures as model of the human intestinal mucosa. Toxicol. Lett..

[27] Wang Y., Yuan L., Yao C., Ding L., Li C., Fang J., Sui K., Liu Y., Wu M. (2014). A combined toxicity study of zinc oxide nanoparticles and vitamin C in food additives. Nanoscale.

[28] Mitchell M.J., Billingsley M.M., Haley R.M., Wechsler M.E., Peppas N.A., Langer R. (2021). Engineering precision nanoparticles for drug delivery. Nat. Rev. Drug Discov..

[29] Cao S.-J., Xu S., Wang H.-M., Ling Y., Dong J., Xia R.-D., Sun X.-H. (2019). Nanoparticles: Oral Delivery for Protein and Peptide Drugs. AAPS PharmSciTech.

[30] Peters R., Kramer E., Oomen A.G., Rivera Z.E.H., Oegema G., Tromp P.C., Fokkink R., Rietveld A., Marvin H.J.P., Weigel S. (2012). Presence of Nano-Sized Silica during In Vitro Digestion of Foods Containing Silica as a Food Additive. ACS Nano.

[31] Walczak A.P., Fokkink R., Peters R., Tromp P., Rivera Z.E.H., Rietjens I.M., Hendriksen P., Bouwmeester H. (2012). Behaviour of silver nanoparticles and silver ions in an in vitro human gastrointestinal digestion model. Nanotoxicology.

[32] Sieg H., Kästner C., Krause B., Meyer T., Burel A., Böhmert L., Lichtenstein D., Jungnickel H., Tentschert J., Laux P. (2017). Impact of an Artificial Digestion Procedure on Aluminum-Containing Nanomaterials. Langmuir.

[33] Zhang Z., Zhang R., Xiao H., Bhattacharya K., Bitounis D., Demokritou P., McClements D.J. (2019). Development of a standardized food model for studying the impact of food matrix effects on the gastrointestinal fate and toxicity of ingested nanomaterials. NanoImpact.

[34] Zhou P., Guo M., Cui X. (2021). Effect of food on orally-ingested titanium dioxide and zinc oxide nanoparticle behaviors in simulated digestive tract. Chemosphere.

[35] Cao X., Zhang T., DeLoid G.M., Gaffrey M.J., Weitz K.K., Thrall B.D., Qian W.-J., Demokritou P. (2020). Evaluation of the cytotoxic and cellular proteome impacts of food-grade TiO2 (E171) using simulated gastrointestinal digestions and a tri-culture small intestinal epithelial model. NanoImpact.

[36] Marucco A., Prono M., Beal D., Alasonati E., Fisicaro P., Bergamaschi E., Carriere M., Fenoglio I. (2020). Biotransformation of Food-Grade and Nanometric TiO_2_ in the Oral–Gastro–Intestinal Tract: Driving Forces and Effect on the Toxicity toward Intestinal Epithelial Cells. Nanomaterials.

[37] Albanese A., Chan W.C. (2011). Effect of Gold Nanoparticle Aggregation on Cell Uptake and Toxicity. ACS Nano.

[38] Mulenos M.R., Liu J., Lujan H., Guo B., Lichtfouse E., Sharma V.K., Sayes C.M. (2020). Copper, silver, and titania nanoparticles do not release ions under anoxic conditions and release only minute ion levels under oxic conditions in water: Evidence for the low toxicity of nanoparticles. Environ. Chem. Lett..

[39] Shalimar D.M., Das P., Sreenivas V., Gupta S.D., Panda S.K., Makharia G.K. (2013). Mechanism of Villous Atrophy in Celiac Disease: Role of Apoptosis and Epithelial Regeneration. Arch. Pathol. Lab. Med..

[40] Sander G.R., Cummins A.G., Powell B.C. (2005). Rapid disruption of intestinal barrier function by gliadin involves altered expression of apical junctional proteins. FEBS Lett..

[41] Yao M., He L., McClements D.J., Xiao H. (2015). Uptake of Gold Nanoparticles by Intestinal Epithelial Cells: Impact of Particle Size on Their Absorption, Accumulation, and Toxicity. J. Agric. Food Chem..

[42] Peters R.J.B., Van Bemmel G., Herrera-Rivera Z., Helsper H.P.F.G., Marvin H.J.P., Weigel S., Tromp P.C., Oomen A.G., Rietveld A.G., Bouwmeester H. (2014). Characterization of Titanium Dioxide Nanoparticles in Food Products: Analytical Methods To Define Nanoparticles. J. Agric. Food Chem..

[43] EFSA Panel on Food Additives and Nutrient Sources added to Food (ANS) (2016). Re-evaluation of titanium dioxide (E 171) as a food additive. EFSA J..

[44] Coméra C., Cartier C., Gaultier E., Catrice O., Panouille Q., El Hamdi S., Tirez K., Nelissen I., Théodorou V., Houdeau E. (2020). Jejunal villus absorption and paracellular tight junction permeability are major routes for early intestinal uptake of food-grade TiO2 particles: An in vivo and ex vivo study in mice. Part. Fibre Toxicol..

[45] Luo Y.-H., Chang L.W., Lin P. (2015). Metal-Based Nanoparticles and the Immune System: Activation, Inflammation, and Potential Applications. BioMed Res. Int..

[46] Bettini S., Boutet-Robinet E., Cartier C., Coméra C., Gaultier E., Dupuy J., Naud N., Taché S., Grysan P., Reguer S. (2017). Food-grade TiO_2_ impairs intestinal and systemic immune homeostasis, initiates preneoplastic lesions and promotes aberrant crypt development in the rat colon. Sci. Rep..

[47] Gillois K., Lévêque M., Théodorou V., Robert H., Mercier-Bonin M. (2018). Mucus: An Underestimated Gut Target for Environmental Pollutants and Food Additives. Microorganisms.

[48] Ghebretatios M., Schaly S., Prakash S. (2021). Nanoparticles in the Food Industry and Their Impact on Human Gut Microbiome and Diseases. Int. J. Mol. Sci..

[49] Gokulan K., Williams K., Orr S., Khare S. (2020). Human Intestinal Tissue Explant Exposure to Silver Nanoparticles Reveals Sex Dependent Alterations in Inflammatory Responses and Epithelial Cell Permeability. Int. J. Mol. Sci..

[50] Schanen B.C., Karakoti A.S., Seal S., Iii D.R.D., Warren W.L., Self W.T. (2009). Exposure to Titanium Dioxide Nanomaterials Provokes Inflammation of an in Vitro Human Immune Construct. ACS Nano.

[51] Galbiati V., Cornaghi L., Gianazza E., Potenza M.A., Donetti E., Marinovich M., Corsini E. (2018). In vitro assessment of silver nanoparticles immunotoxicity. Food Chem. Toxicol..

[52] Yao L., Tang Y., Chen B., Hong W., Xu X., Liu Y., Aguilar Z.P., Xu H. (2020). Oral exposure of titanium oxide nanoparticles induce ileum physical barrier dysfunction via Th1/Th2 imbalance. Environ. Toxicol..

[53] Gross S., Van Wanrooij R.L., Nijeboer P., Gelderman K.A., Cillessen S.A.G.M., Meijer G.A., Mulder C.J.J., Bouma G., Von Blomberg B.M.E., Bontkes H.J. (2013). Differential IL-13 Production by Small Intestinal Leukocytes in Active Coeliac Disease versus Refractory Coeliac Disease. Mediat. Inflamm..

[54] Abdukhakimova D., Dossybayeva K., Poddighe D. (2021). Fecal and Duodenal Microbiota in Pediatric Celiac Disease. Front. Pediatr..

[55] Valitutti F., Cucchiara S., Fasano A. (2019). Celiac Disease and the Microbiome. Nutrients.

[56] Lomer M.C.E., Thompson R.P.H., Powell J.J. (2002). Fine and ultrafine particles of the diet: Influence on the mucosal immune response and association with Crohn’s disease. Proc. Nutr. Soc..

[57] Lacerda S.H.D.P., Park J.J., Meuse C., Pristinski D., Becker M.L., Karim A., Douglas J.F. (2009). Interaction of Gold Nanoparticles with Common Human Blood Proteins. ACS Nano.

[58] Frazer A., Fletcher R., Shaw B., Ross C., Sammons H., Schneider R. (1959). Gluten-Induced Enteropathy The Effect Of Partially Digested Gluten. Lancet.

[59] Mancuso C., Barisani D. (2019). Food additives can act as triggering factors in celiac disease: Current knowledge based on a critical review of the literature. World J. Clin. Cases.

