# Supplementary material for: Dietary Nanoparticles Interact with Gluten Peptides and Alter the Intestinal Homeostasis Increasing the Risk of Celiac Disease
Source: Int J Mol Sci. 2021 Jun 5;22(11):6102. doi: 10.3390/ijms22116102 (PMC8201331; doi:10.3390/ijms22116102)
Supplement: Supplementary file 1 [file ijms-22-06102-s001.zip › ijms-1204678-supplementary.pdf]

Article

Dietary nanoparticles interact with gluten peptides and alter the intestinal homeostasis increasing the risk of Celiac Disease

Clara Mancuso [1,2], Francesca Re[1], Ilaria Rivolta[1], Luca Elli[3], Elisa Gnodi[1], Jean-François Beaulieu[2], Donatella Barisani[1,*]

[1] School of Medicine and Surgery, University of Milano-Bicocca, 20900 Monza, Italy; clara.mancuso@unimib.it , francesca.re1@unimib.it , ilaria.rivolta@unimib.it , e.gnodi@campus.unimib.it
[2] Laboratory of Intestinal Physiopathology, Faculty of Medicine and Health Sciences, Université de Sherbrooke, Sherbrooke, J1H 5N4, QC, Canada; Jean-Francois.Beaulieu@usherbrooke.ca
[3] Centre for the Prevention and Diagnosis of Celiac Disease, Gastroenterology and Endoscopy Unit, Fondazione IRCCS Ca' Granda Ospedale Maggiore Policlinico, 20122 Milan, Italy; luca.elli@policlinico.mi.it
* Correspondence donatella.barisani@unimib.it ; Tel.: +39-02-64488304

Supplementary Materials

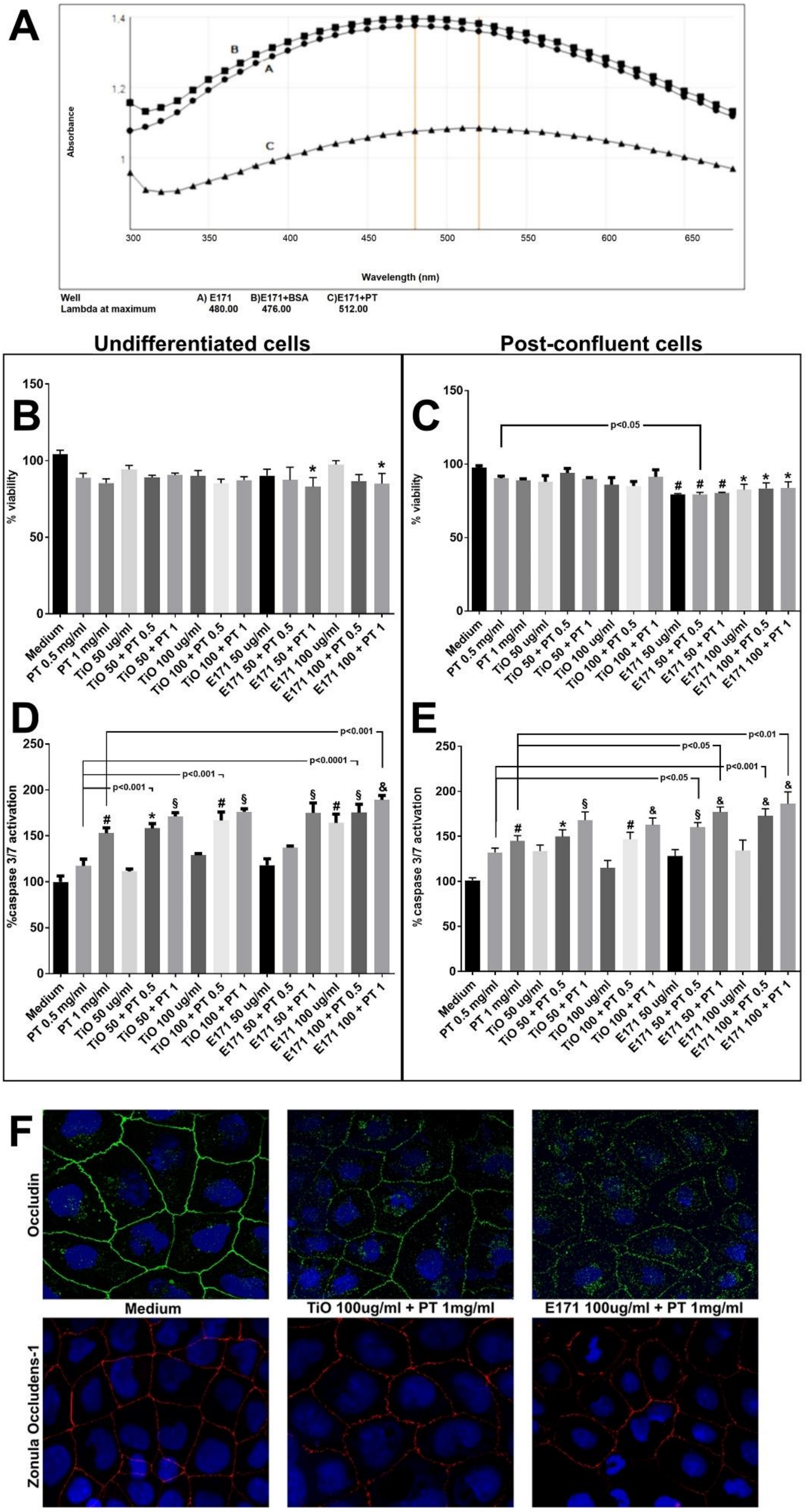
A
Absorbance
Wavelength (nm)
Well
Lambda at maximum
A) E171 480.00
B)E171+BSA 476.00
C)E171+PT 512.00
Undifferentiated cells
Post-confluent cells
B
C
% viability
p<0.05
Medium
PT 0.5 mg/ml
PT 1 mg/ml
TiO 50 ug/ml
TiO 50 + PT 0.5
TiO 50 + PT 1
TiO 100 ug/ml
TiO 100 + PT 0.5
TiO 100 + PT 1
E171 50 ug/ml
E171 50 + PT 0.5
E171 50 + PT 1
E171 100 ug/ml
E171 100 + PT 0.5
E171 100 + PT 1
D
E
%caspase 3/7 activation
% caspase 3/7 activation
p<0.001
p<0.0001
p<0.01
p<0.05
F
Occludin
Zonula Occludens-1
Medium
TiO 100ug/ml + PT 1mg/ml
E171 100ug/ml + PT 1mg/ml

Figure S1: E171 vs TiO_2NP. (A) UV-Vis spectra of E171; (B) MTT performed on undifferentiated Caco-2 cells; (C) MTT performed on post-confluent Caco-2 cells; (D) Apoptosis Assay on undifferentiated Caco-2 cells; (E) Apoptosis Assay on post-confluent Caco2 cells; Significance versus untreated cells is indicated above each column (*p<0.05; #p<0.01; §p<0.001; &p<0.0001), whereas versus PT is represented by bars. Data are shown as mean value and SD. (F) OCLN and ZO-1 immunofluorescence in post-confluent Caco2 cells. We only reported images of cells treated with the combinations: TiO_2NP (100ug/ml) +PT (1mg/ml) and E171 (100ug/ml) + PT (1mg/ml).

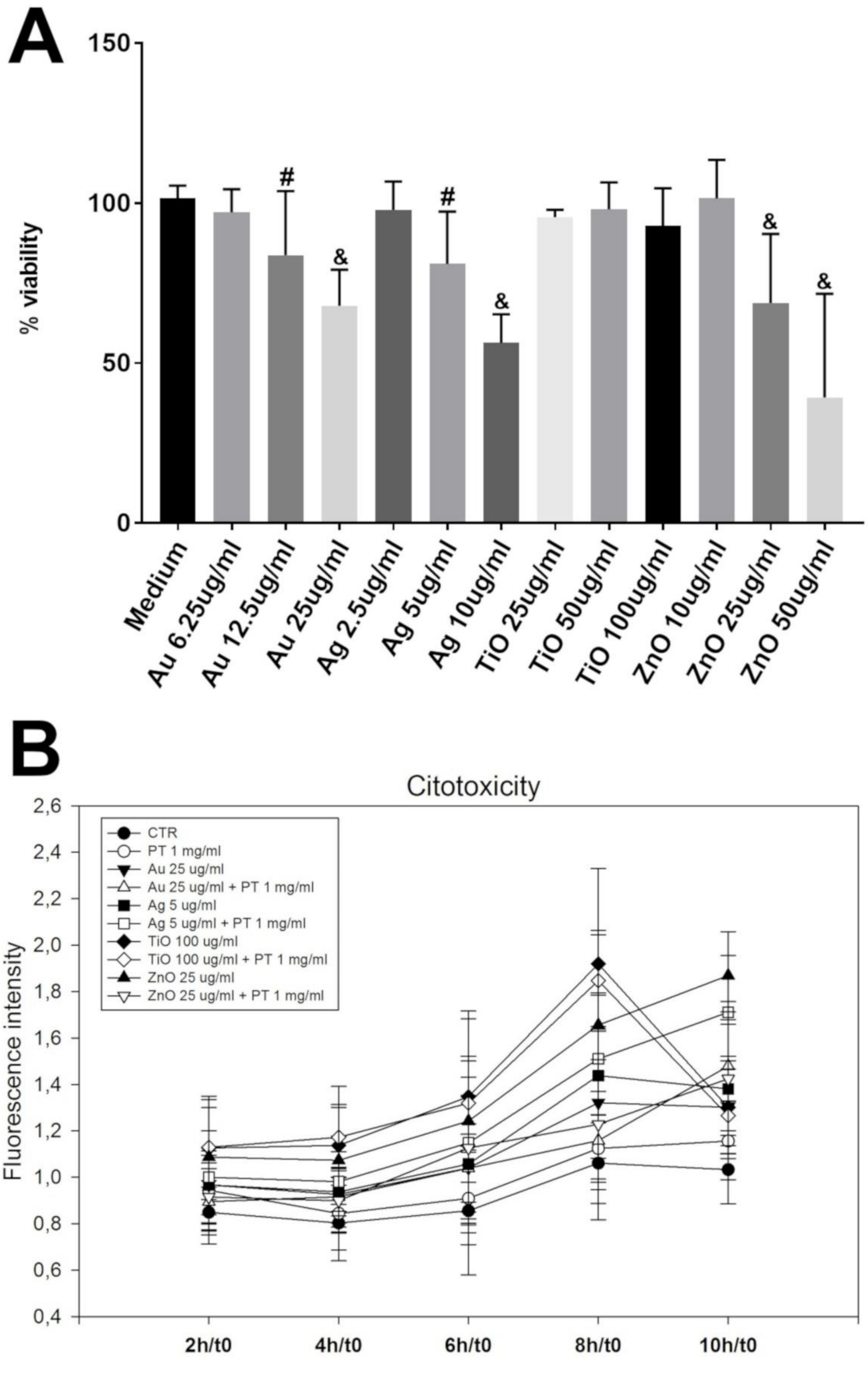

***Figure S2*:** Dose response of the mNPs and time course of the various treatments in undifferentiated Caco2 cells. (A) MTT assay at 24 hours. Data are expressed as % of untreated cells (Medium). Significance versus untreated cells is indicated above each column (#p<0.01; &p<0.0001). (B) Time course of cytotoxicity caused by the various treatments. The fluorescence intensity represented is the one detected at the single time point minus the fluorescence at time 0.

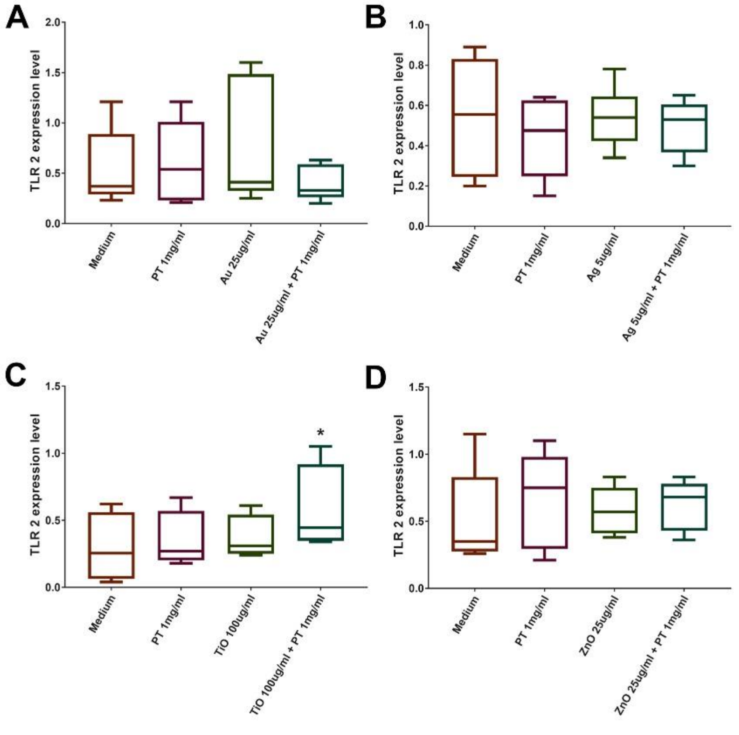

Figure S3. TLR2 expression in CeD patients on a GFD. Duodenal biopsies stimulated 4h with PT 1mg/ml ± (A) AuNP 25 ug/ml (n=5); (B) AgNP 5 ug/ml (n=6); (C) TiO2NP 100 ug/ml (n=4); (D) ZnONP 25 ug/ml (n=5). Significance versus untreated biopsies is indicated above each column (*$p<0.05$). Box plots represent median, 25th, and 75th percentiles. Whiskers indicate 5th and 95th percentiles.

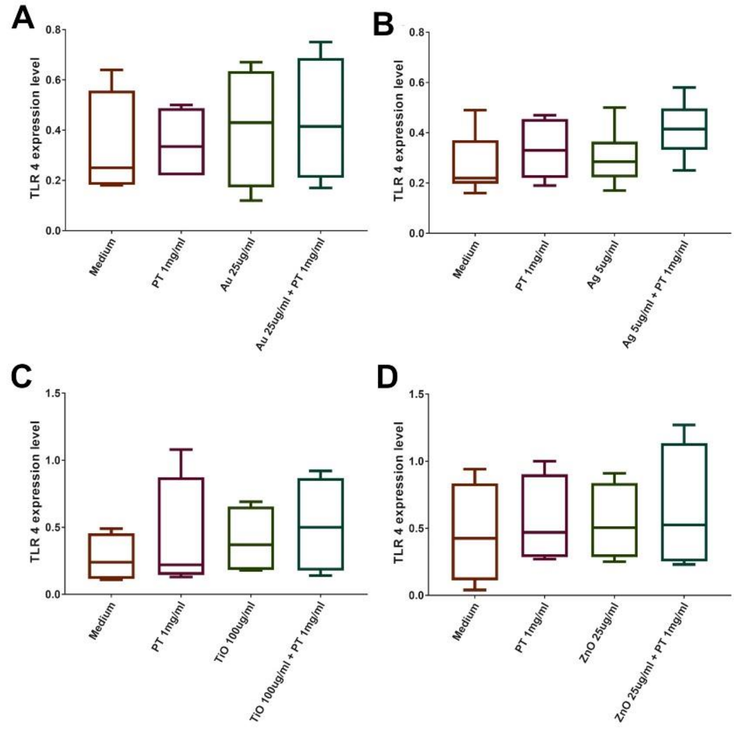

Figure S4. TLR4 expression in CeD patients on a GFD. Duodenal biopsies stimulated 4h with PT 1mg/ml ± (A) AuNP 25 ug/ml (n=5); (B) AgNP 5 ug/ml (n=6); (C) TiO2NP 100 ug/ml (n=4); (D) ZnONP 25 ug/ml (n=5). Significance versus

untreated biopsies is indicated above each column (*$p<0.05$). Box plots represent median, 25th, and 75th percentiles. Whiskers indicate 5th and 95th percentiles.
